# Supplementary material for: Long decay length of magnon-polarons in BiFeO3/La0.67Sr0.33MnO3 heterostructures
Source: Nat Commun. 2021 Dec 14;12:7258. doi: 10.1038/s41467-021-27405-2 (PMC8671416; doi:10.1038/s41467-021-27405-2)
Supplement: Supplementary file 1 — Supplementary Information [file 41467_2021_27405_MOESM1_ESM.pdf]

Long decay length of magnon-polarons in $BiFeO_3/La_{0.67}Sr_{0.33}MnO_3$ heterostructures

I. RSM and TEM characterization

Before we pattern the sample with spin-wave antennas, we characterized the film with reciprocal space mappings (RSM) to obtain the lattice constant and the sample growth state. The RSM result is displayed in Fig. S1**a**. From the RSM spectra, the calculated in-plane lattice constant of *a* axis is 3.859 Å for NGO, LSMO, and BFO. The out of plane lattice constant of *c* axis is 3.855 Å for NGO, 3.890 Å for LSMO, and 4.171 Å for BFO. Besides, we characterized the BFO/LSMO/NGO structure with energy-dispersive X-ray spectroscopy (EDS), the results are displayed in Fig. S1**b** to **h**. Both the RSM and TEM characterization show a good epitaxy of the film.

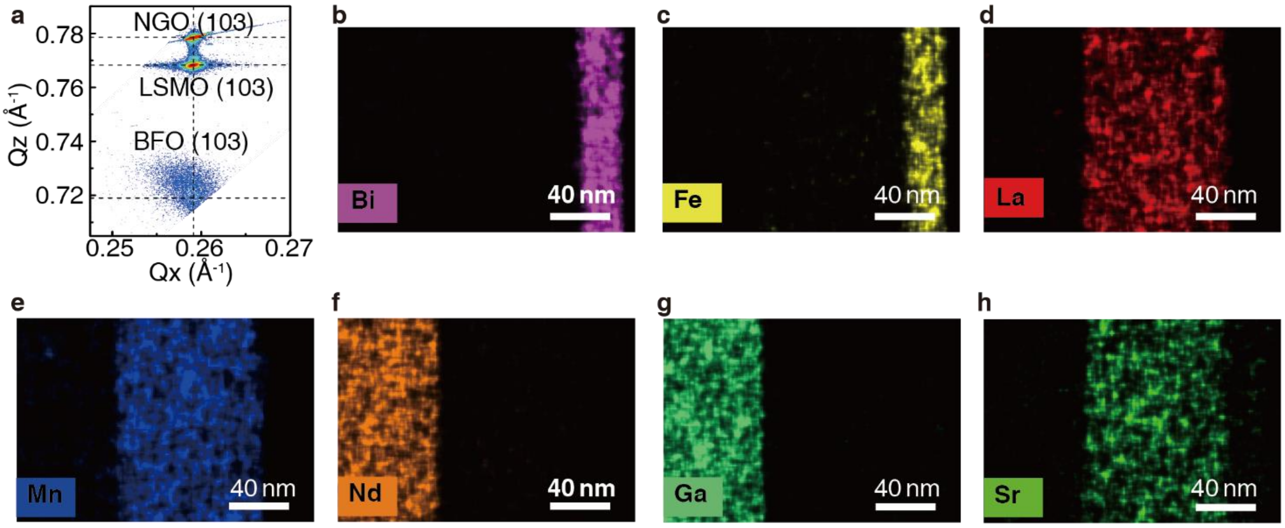

Fig. S1. Reciprocal space mappings characterization X-ray diffraction and energy dispersive x-ray spectroscopy characterization. a, The crystal structures of thin film were characterized by RSM around the NGO (103) diffraction spot. The diffraction spots of LSMO and BFO clearly show the good epitaxy of these two layers. **b-h**, the elements bismuth, iron, lanthanum and manganese, are displayed in different color. The scale bars in **b-h** are 40 nm.

II. Detailed information of devices with different antennas

Fig. S2**a-c** show SEM images of the antennas used for spin-wave excitation and detection, the BFO/LSMO samples are patterned into mesas with chamfer ends as denoted by the blue part to reduce spin-wave reflections from the edges, and **d** to **f** are the corresponding zoom-in images, the widths of the nano-stripline (NSL) antennas with orange color are denoted in each figure. By connecting these antennas with the VNA through microwave probes, rf currents can be sent into the antenna. Spin waves are excited due to the changing of magnetic flux from the rf magnetic fields. The wave vector distribution of the excited spin waves depending on the design of the antennas can be obtained by a Fourier transformation. Fig. S2**g-i** show the Fourier transformation of the antenna excitation of **d-f**.

Broadband wavevector distributions can be observed in NSL antennas while discrete wavevector distribution is observed for the meander CPW antenna. It is worthy to note that the third peak ($k = 16$ rad μm^{-1}) in **i** corresponds to the $\lambda = 390$ nm spin-wave wavelength, which is 20 times smaller than the value reported for surface acoustic waves with wavelength $\lambda \sim 8$ μm (Ref. 26 in the main text).

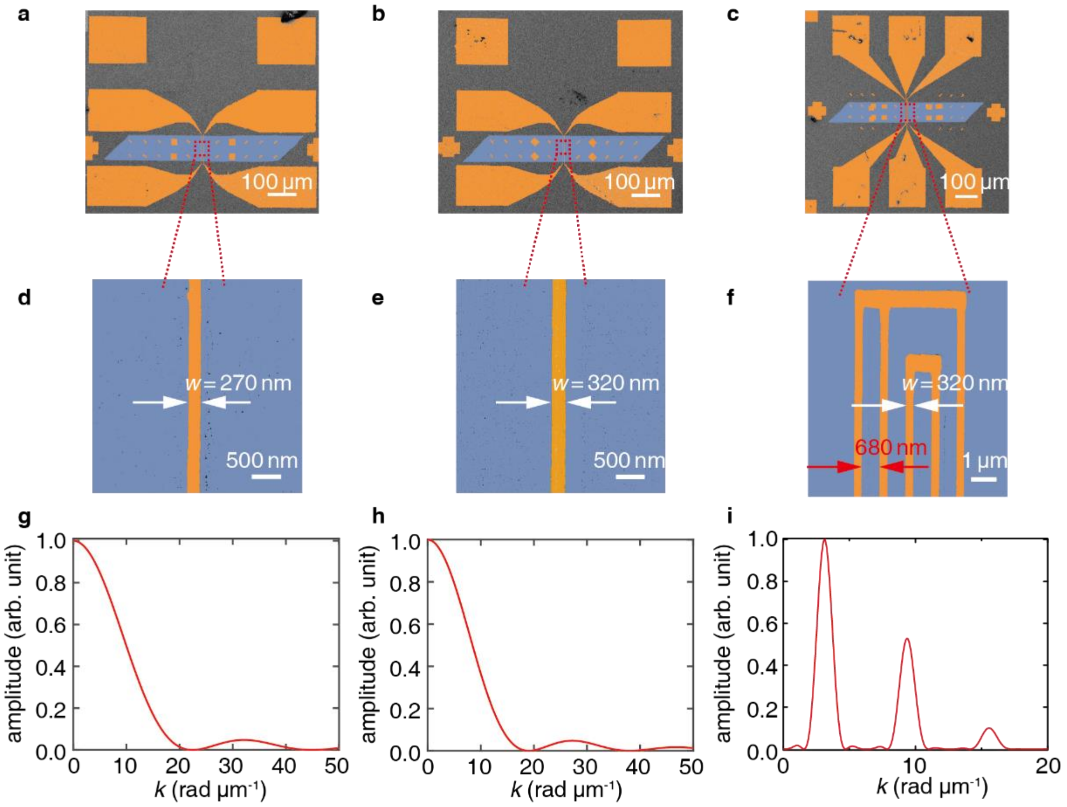

Fig. S2. Information of different antennas. a-f, SEM images of the antennas and their zoom-in images used for spin-wave excitation and detection. The scale bars are 100 μm in **a-c**, 500 nm in **d** and **e,** and 1 μm in **f**, the widths and gaps between the antenna lines are denoted in each figure. **g-i**, The Fourier transformations of the antennas in **d-f**, from which we can see that the antennas of different sizes and designs possess different k (wavevector) distribution.

There are two different chips patterned with spin-wave devices, Sample Z mainly patterned with the NSL antennas with different spin-wave propagation distances (below 300 μm) at $\beta = 0°$, and different wave vector orientations at $d = 6$ μm; Sample N is patterned with meander CPWs of ultra-long propagation distances over 1 mm at $\beta = 0°$. The device information is listed in Table 1.

Table 1 | Parameters and properties of different devices

Sample No.	β [deg]	d [μm]	Antenna type	k [rad μm^{-1}]	v_g [km s^{-1}]	S_{21} Mode Y	S_{21} Mode X
Z01	0	2 μm	NSL	9.0	0.80	5.56	1.33
Z02	0	6 μm	NSL	11.6	0.84	1.95	1.22
Z03	30	6 μm	NSL	8.3	0.87	1.10	N/A
Z04	45	6 μm	NSL	11.6	0.87	1.76	N/A
Z05	60	6 μm	NSL	9.5	0.84	1.33	N/A
Z06	90	6 μm	NSL	11.6	0.78	1.22	N/A
Z07	0	12 μm	NSL	9.8	0.96	0.58	0.81
Z08	0	20 μm	NSL	11.6	0.90	0.10	0.88
Z09	0	24 μm	NSL	5.9	0.96	0.07	1.02
Z10	0	30 μm	NSL	10.4	N/A	N/A	0.26
Z11	0	50 μm	NSL	9.8	N/A	N/A	0.27
Z12	0	100 μm	NSL	7.9	N/A	N/A	0.09
Z13	0	150 μm	NSL	26.2	N/A	N/A	0.09
Z14	0	300 μm	NSL	7.85	N/A	N/A	0.067
Z15	0	12 μm	CPW	3.1	1.14	0.50	N/A
N01	0	20 μm	CPW	3.1	N/A	N/A	0.130
N02	0	50 μm	CPW	3.1	N/A	N/A	0.097
N03	0	100 μm	CPW	3.1	N/A	N/A	0.09
N04	0	940 μm	CPW	3.1	N/A	N/A	0.047
N05	0	1060 μm	CPW	3.1	N/A	N/A	0.044

The wavevector angle with respect to [110] direction of LSMO crystal (β), propagation distance (d), group velocity (v_g) at 100 mT for Sample Z. Estimated wavevector (k) by FFT of antenna. The CPW used in this work is the meander-like CPW as shown in Fig. S2**f**.

III. Magnetic damping characterization

To characterize the basic magnetic property of Sample Z, we did the ferromagnetic resonance (FMR) measurement to determine its magnetic damping, and the field derivative of the FMR lines and the corresponding fitting are listed in Fig. S3**a**. The linear fitting of the FMR linewidths at different frequencies is shown in Fig. S3**b** and the yielded damping is 1.2×10^{-3}.

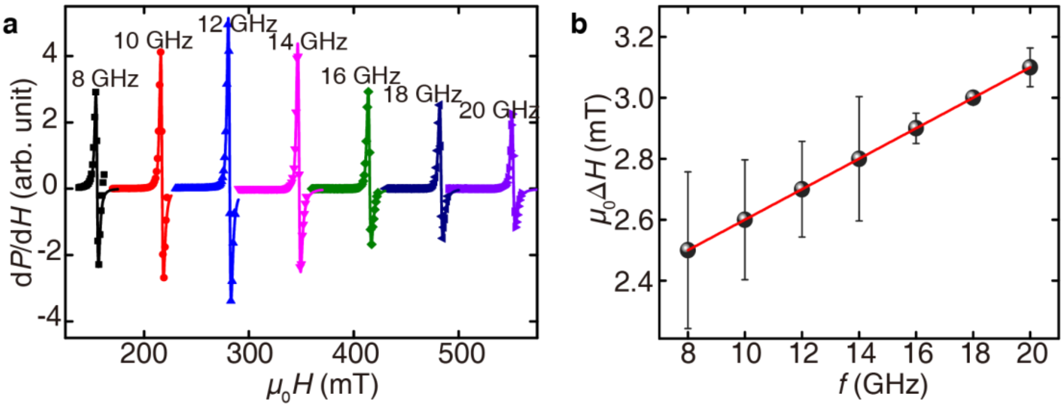

Fig. S3. Magnetic damping characterization on Sample Z. a, Ferromagnetic resonance (FMR) taken at different frequencies (dots) and the corresponding fittings (solid lines). **b**, FMR linewidths extracted from the field-dependent measurements plotted as a function of frequency. The fitted slope yields a damping $\alpha = 1.2 \times 10^{-3}$.

IV. Bare LSMO sample without BFO capping layer

To investigate the origin of mode X, we did the angle-resolved spin wave measurement on the bare LSMO film without BFO capping layer on top. The NSL antennas are 2 μm away from each other. From the measurement result, we can observe that without BFO capping layer on top of LSMO, mode B, which we attribute to the BFO/LSMO interface, is gone, as a result, the hybridized mode X induced by the coupling between the piezoelectric BFO and magnetic LSMO is also absent.

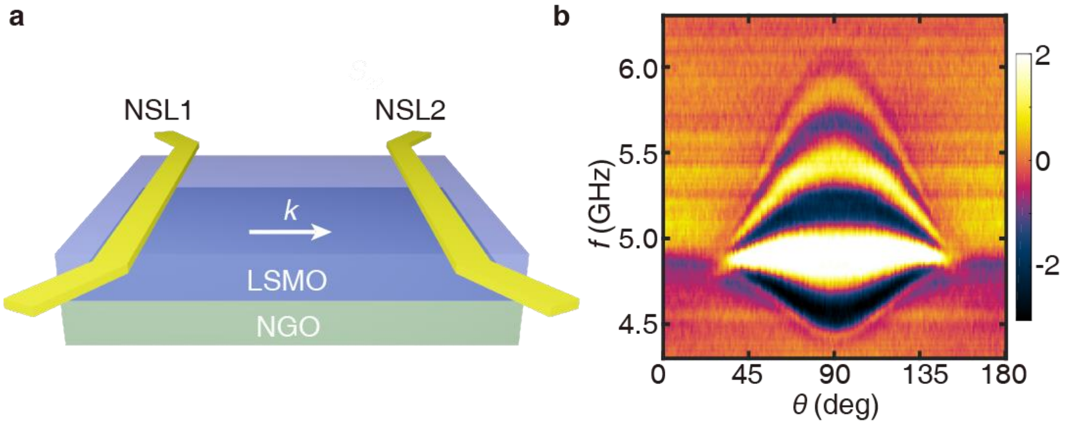

Fig. S4. Measurements on a bare LSMO sample without BFO. a, Schematic of the spin-wave measurement configuration on a bare LSMO sample. Two identical nano-stripline (NSL) antennas of 6 μm apart are fabricated on top of the bare LSMO sample, the wavevector k is parallel to the [110] crystalline orientation of LSMO. **b**, The angle-resolved spin-wave transmission spectra S_{21} measured on the bare LSMO sample. The rotating field magnitude is fixed at 50 mT. The LSMO film thickness is 20 nm. The microwave power is set at -10 dBm.

V. Theoretical modeling

To show that magnon-phonon coupling can reproduce most of the features observed in the present experiments, we consider a model depicted in Fig. S5. We focus on the interaction between a mode of phonon and a mode of magnon with a common in-plane wavevector k. The important assumptions and the rational behind them are as follows:

1. We assume that the phonon is localized and uniform across the thickness direction in the BFO layer. The justification comes from the fact that mode X vanishes in the transmission spectra on a bare LSMO film without BFO (Fig. S4). With a significant phonon velocity mismatch in BFO (Refs. 25, 33, 46 in the main text) and LSMO [1], the phonon mode cannot penetrate efficiently into LSMO and the coupled elastic wave is expected to be evanescent into the LSMO layer. This is analogous to the Love wave. While the nature of the evanescent decay into LSMO is not known, we simply set its amplitude zero in LSMO. To compensate this, we have introduced the fictitious overlap layer of an unspecified thickness $t_{\mathrm{Interface}}$ between the two layers.
2. We assume that the LA phonon dispersion and its magneto-elastic coupling to the LSMO magnon are both isotropic. This is purely for simplicity, but turns out to work well.
3. We assume that the LSMO magnon is described by the thin-film approximation for ferromagnetic spin waves [2]. Note that the approximation is based on neglecting the spatial variation in the thickness direction. This implies that the magnetic properties of BFO are largely left unaccounted for in this model. It can be partially justified since the bulk BFO spin waves should have higher frequencies than those of LSMO, given the antiferromagnetism and high transition temperature. While there are claims of an interface ferromagnetism (Ref. 31 and 32 in the main text), its nature is unclear at this stage and we are unable to model it in a tractable manner.

The model is admittedly oversimplified and not meant to explain the data quantitatively. For that, nevertheless, the qualitative agreement is surprisingly good, which offers an a posteriori justification of the made simplifications.

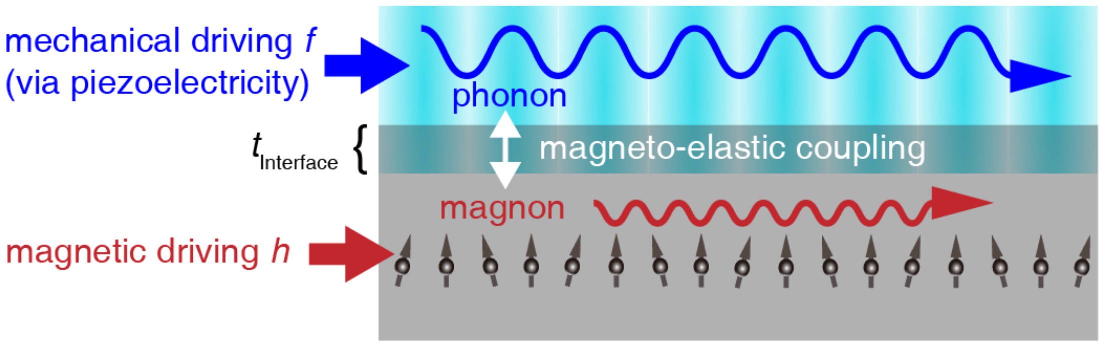

Fig. S5. The schematic of the theoretical model studied in this work. The system consitsts of two layers: The top layer hosts a longitudinal acoustic wave mode whose properties are assumed to coincide with those of the bulk BFO LA mode. The bottom layer is a simple ferromagnet with an in-plane uniaxial anisotropy. To phenomenologically parameterize the interfacial interaction between the layers, we introduce an overlap region of thickness $t_{\mathrm{Interface}}$, which is a free parameter.

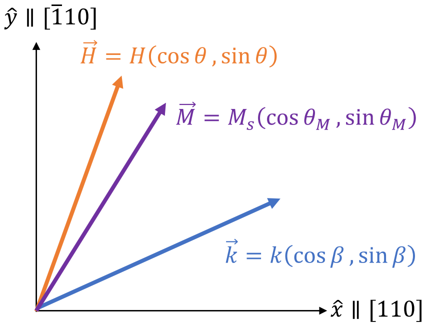

Fig. S6. The in-plane geometry of the theoretical model.

Let us denote the in-plane static magnetic field by $\vec{H} = H(\cos\theta, \sin\theta)$ where θ is measured with respect to the crystalline [110] direction. We introduce an in-plane easy-axis anisotropy $K = 4000\ \mathrm{J\,m^{-3}}$ as observed in the spin-wave spectrum, which implies that the angle of the direction of the equilibrium magnetization $\vec{M} = M_s(\cos\theta_M, \sin\theta_M)$ in general differs from $\vec{H}$. In the model, the axis of wave propagation can also be tilted from the [110] direction, which is denoted by β. The wavevector along the axis is parametrized by $k \in \mathbb{R}$. See Fig. S6.

The dynamics of the magneto-elastic waves in the present setup is described by (Ref. 39 in the main text)

$$\begin{pmatrix} i\alpha M_s\omega/\gamma - F_{zz} & iM_s\omega/\gamma & 0 \\ -iM_s\omega/\gamma & i\alpha M_s\omega/\gamma - F_{\parallel} & -2g t_{\mathrm{Interface}}/t_{\mathrm{FM}} \\ 0 & -2\bar{g} t_{\mathrm{Interface}}/t_{\mathrm{AFM}} & \rho(\omega^2/k^2 - c_s^2 + i\omega/\tau k^2) \end{pmatrix} \begin{pmatrix} n_z \\ n_{\parallel} \\ ku \end{pmatrix} = \begin{pmatrix} -\mu_0 M_s h \\ 0 \\ f/k \end{pmatrix}, \quad \text{(S1)}$$

where $n_z, n_{\parallel}$ are out-of-plane and in-plane components of the normalized magnetization respectively, u is the elastic deformation along $\vec{k}$, $\gamma = 2\pi \times 29\ \mathrm{GHz\,T^{-1}}$ is the gyromagnetic ratio, μ_0 is permeability of vacuum, α is the Gilbert damping constant, τ is the frictional relaxation time for phonons, t_{FM} and t_{AFM} are the thicknesses of the LSMO and BFO layers respectively, h is the out-of-plane component of the driving microwave magnetic field, the in-plane component is set zero for simplicity, and f is the amplitude of the mechanical force density generated by the NSL or CPW via piezoelectric effect of BFO. The remaining symbols $F_{zz}, F_{\parallel}, g$ are defined by

$$F_{zz} = \mu_0 M_s H\cos(\theta - \theta_M) + \mu_0 M_s^2 \frac{1 - e^{-|k|t_{\mathrm{FM}}}}{|k|t_{\mathrm{FM}}} + Ak^2 - K\sin^2\theta_M + B,$$

$$F_{\parallel} = \mu_0 M_s H\cos(\theta - \theta_M) + \mu_0 M_s^2 \left(1 - \frac{1 - e^{-|k|t_{\mathrm{FM}}}}{|k|t_{\mathrm{FM}}}\right)\sin^2(\beta - \theta_M) + Ak^2 + K\cos 2\theta_M + B,$$

$$g = ib\sin 2(\beta - \theta_M), \qquad \text{(S2)}$$

where A is the exchange constant in the LSMO layer, B is an offset fitting parameter, and b is the bare magneto-elastic coupling in the interface region. The equation has been Fourier transformed in time and space, and is written in the unit of energy density.

Most of the model parameters can be inferred from the available data or literature values, e.g. $A = 2 \times 10^{-12}\ \mathrm{J\,m^{-1}}$. We take $B = 10^4\ \mathrm{J\,m^{-3}}$, $t_{\mathrm{FM}} = 80\ \mathrm{nm}$ and $M_s = 276\ \mathrm{kA\,m^{-1}}$, which reproduces mode Y reasonably well. The relaxation parameters are set $\alpha = 2 \times 10^{-3}, \tau = 10^{-7}\ \mathrm{s}$ throughout. Although these might not be the best-fit values, we do not aim to be quantitatively correct here. Thus

the main unknowns are the magneto-elastic coupling b and the fictitious interface thickness $t_{\text{Interface}}$. It is instructive to rescale the dimensionless variables $n_z, n_\parallel$ and u as well as the driving fields h, f, which can integrate the two into a single effective coupling parameter b_{eff} yielding

$$\chi_k^{-1}\begin{pmatrix} n_z \\ n_\parallel \\ ku \end{pmatrix} = \begin{pmatrix} -\mu_0 M_s h \\ 0 \\ f/k \end{pmatrix}, \quad \text{(S3)}$$

$$\chi_k^{-1} = \begin{pmatrix} i\alpha M_s\omega/\gamma - F_{zz} & iM_s\omega/\gamma & 0 \\ -iM_s\omega/\gamma & i\alpha M_s\omega/\gamma - F_\parallel & -2ib_{\text{eff}}\sin 2(\beta-\theta_M) \\ 0 & 2ib_{\text{eff}}\sin 2(\beta-\theta_M) & \rho(\omega^2/k^2 - c_s^2 + i\omega/\tau k^2) \end{pmatrix},$$

where

$$b_{\text{eff}} = b\frac{t_{\text{Interface}}}{\sqrt{t_{\text{FM}}t_{\text{AFM}}}}. \quad \text{(S4)}$$

This reflects the obvious fact that the effective coupling should be smaller than the microscopically determined bare coupling b since the spin waves mainly live in LSMO while the acoustic waves are in BFO so that the two can interact only in a small region near the interface.

To simulate the NSL or CPW measurements, we first specify h and f as functions of k. For modeling the NSL with the width $w = 300$ nm, we take

$$h_k = h_0 e^{-k^2w^2/(2\pi)^2}, \qquad f_k = f_0 e^{-k^2w^2/(2\pi)^2}. \quad \text{(S5)}$$

The constant factors depend on the detailed setup of the measurements and we are unable to estimate their values. In the simulations, we fixed $h_0 = 1$ and varied f_0 to improve the agreement with the experimental data, by which we found that the result is insensitive to f_0 at least up to $f_0 = 10000$. The oscillating magnetic field underneath the receiving NSL or CPW should be proportional to

$$n_z(d) = \sum_k (1 \quad 0 \quad 0)\chi_k \begin{pmatrix} -\mu_0 M_s h_k \\ 0 \\ f_k/k \end{pmatrix} e^{ikd},$$

and similarly, the oscillating electric field should be proportional to

$$\epsilon(d) = \sum_k (0 \quad 0 \quad 1)\chi_k \begin{pmatrix} -\mu_0 M_s h_k \\ 0 \\ f_k/k \end{pmatrix} e^{ikd}$$

due to the inverse piezoelectric effect. Although the nonzero width of the NSL or CPW implies a further smearing of the signals by another weight function of k such as those defined in Eq. S5, we ignore this effect for simplicity. The signal picked up by the NSL or CPW is a mixture of the magnetic and electric signals, but it is prohibitively difficult to know how much is attributed to the magnetic or electric signal. In relation to this, we remark that unlike the magnetic signal attributed to mode Y that is subject to the phase oscillation (see mode Y), the signal detected for mode X (most notably due to the phonon peaks in Fig. S13**d**) always has a negative imaginary part only. To account for the latter observation, we calculate

$$s(d) = \text{Re}[n_z(d)] - c|\epsilon(d)|$$

as the theoretical model for the observed signal, where the numerical constant c is adjusted to reproduce experimentally observed features.

The simulation results for $f_0 = 1, c = 6000$ and $d = 0, 2, 6, 12, 24, 50, 100$ μm are given in Fig. S7. The effective magneto-elastic coupling has been set $b_{\text{eff}} = 2 \times 10^5$ J m^{-3}. If this value were taken for the bare coupling b, it would have been considered a typical strength for magnetic oxides. However, referring to Eq. S4, the effective coupling is smaller than the bare coupling by factor of $\frac{t_{\text{Interface}}}{\sqrt{t_{\text{FM}}t_{\text{AFM}}}}$. We have $t_{\text{FM}} = 80$ nm and $t_{\text{AFM}} = 20$ nm so that the reduction factor is $t_{\text{Interface}}$ nm/40.

To obtain $b_{\mathrm{eff}} = 2 \times 10^5\ \mathrm{J\ m^{-3}}$, the bare b should be in the range of 10^6 or 10^7. Note that even if we take into account the evanescent penetration of the BFO phonon into LSMO, that increases both $t_{\mathrm{Interaction}}$ and t_{AFM} so that the effective coupling is always significantly smaller than the bare coupling. This suggests that the bare magneto-elastic coupling of the BFO/LSMO interface is quite large. The modeling here is simplistic, and there could well be other possibilities such as an exotic hybridization of the LSMO magnons with the BFO magnetic dynamics. However, judging from the good agreement of the present simulations with both the modes X and Y, it is difficult to think that the spin-wave dispersion is affected by the BFO layer in any significant way. At this stage, we believe that there is a good evidence that the observed signal and the long decay length are mainly due to the hybridization between the LSMO magnons and the BFO phonons. We do recognize that there are some unexplained features remaining, which offers an exciting prospect for more research into this intriguing heterostructure.

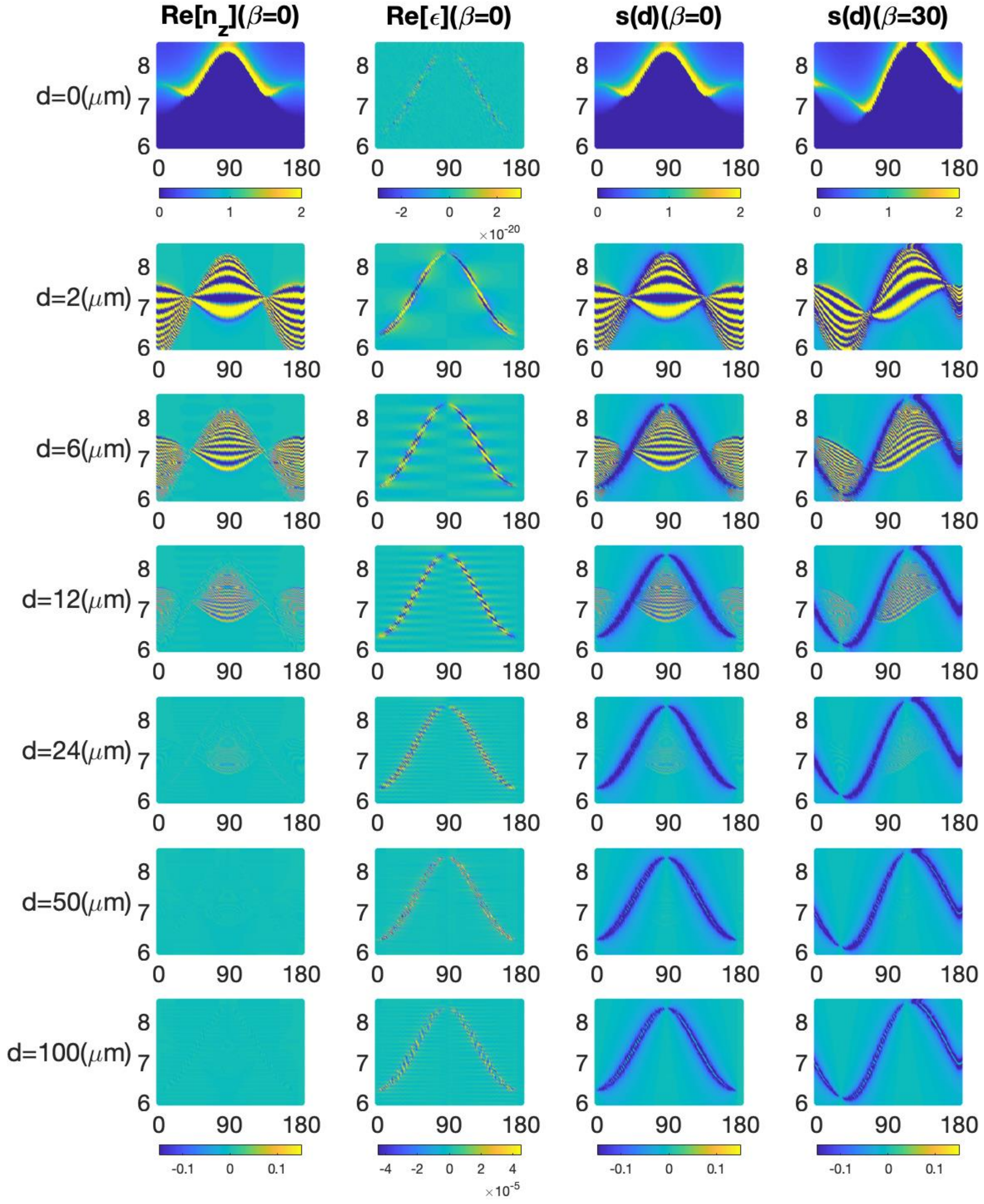

Fig. S7. The numerically calculated $n_z(d), \epsilon(d), s(d)$ as functions of θ and f for different values of d. The horizontal axes indicate θ in degree, and the vertical axes show the frequency f in GHz for all the panels. Left-most column: The real part of $n_z(d)$ for $\beta = 0°$. The color scale is common to all the distances except for $d = 0$

which has its own color bar. Center left column: The real part of $\epsilon(d)$ for $\beta = 0°$. All the panels use the same color scale except for $d = 0$. Note that our simulation predicts the phase sensitive elastic signals, and the results show the interference pattern. Center right column: $s(d)$ for $\beta = 0°$. The color scale for $d = 0$ differs from the rest. The elastic signal appears to split into two strips for $d = 50, 100$ μm, which occurs because the group velocity of the hybridized mode is small at the center of the anti-crossing (see Fig. S8). Right-most column: $s(d)$ for $\beta = 30°$. The pattern qualitatively agrees with the experimental data shown in Fig. S11.

VI. Dispersion relations for magnons and phonons

To help understanding the characteristic shape of the θ-f plots, we plot the eigenfrequencies of the magnons and phonons coupled via Eq. S3 as functions of the wavevector k. They are determined as the roots of $\det(\chi_k^{-1}) = 0$ for $\alpha = \tau^{-1} = 0$, which solves explicitly as

$$2\omega^2 = c_s^2 k^2 + \frac{\gamma^2 F_\parallel F_{zz}}{M_s^2} \pm \sqrt{\left(c_s^2 k^2 - \frac{\gamma^2 F_\parallel F_{zz}}{M_s^2}\right)^2 + \frac{16\gamma^2 F_{zz} k^2 b_{\text{eff}}^2}{\rho M_s^2} \sin^2 2(\beta - \theta_M)}. \quad \text{(S6)}$$

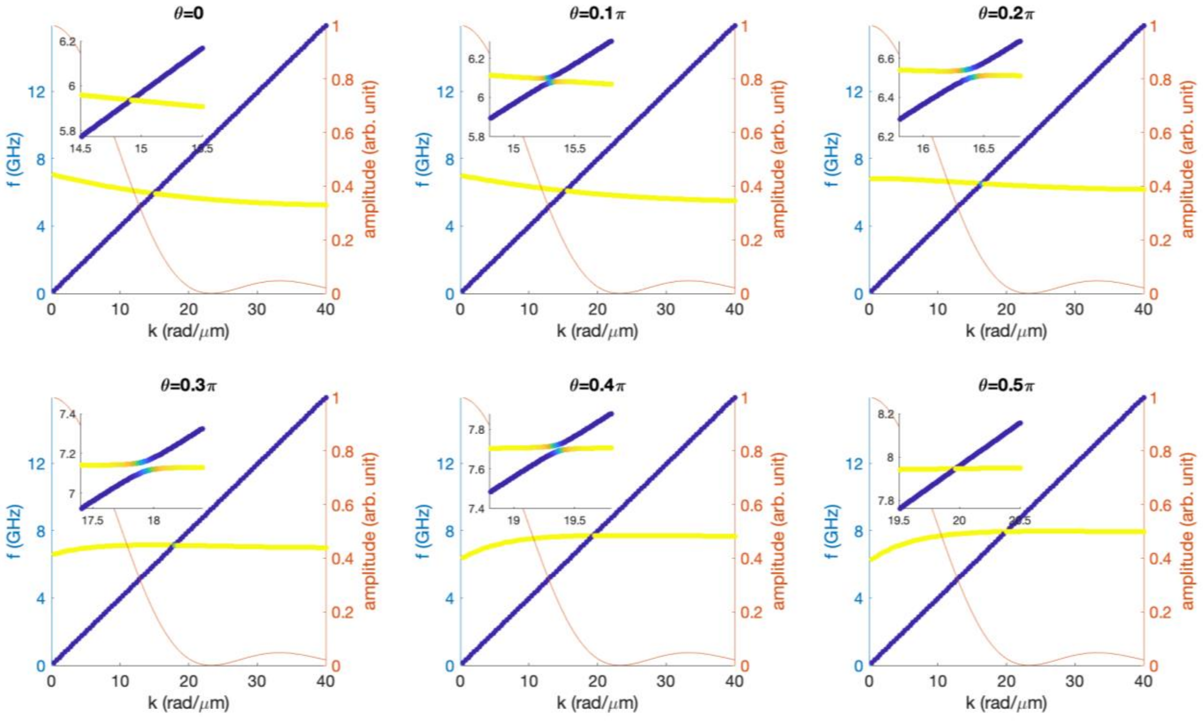

Fig. S8. Dispersion relations for the magnons and phonons for different values of θ with $\beta = 0°$. Commonly to the main panels and insets, the horizontal axes are k in the unit of rad/μm, and the left vertical axes are the frequency in the unit of GHz. The insets show the zoom-in near the magnon-phonon hybridization. The color of thick curves indicates the degree of magnon-phonon hybridization: yellow and dark blue correspond to 100% magnon and phonon respectively while green implies roughly 50-50 hybridization. The red curves, attached to the right vertical axes, reproduce Fig. S2g.

The results for different values of θ are plotted in Fig. S8. The parameter values are the same as those used for the simulations in the previous section. It is noticed that at the chosen value of b_{eff}, the hybridization is still small in the natural scale of the magnetostatic spin waves at GHz range. No anti-crossing is observed for the pure backward volume ($\theta = 0$) and pure Damon-Eshbach ($\theta = \pi/2$)

geometries, which is a direct consequence of the $\sin 2(\beta - \theta_M)$ dependence of the coupling g (Eq. S2). One can observe that the frequency at which the crossing occurs increases as θ increases, which is responsible for the angular dependence of mode X. We also note that around $\theta = \pi/4$, the magnon dispersion becomes almost flat, which gives a large magnon density of states. This is reflected upon the strong enhancement of the S_{22} signal. This phenomenon may contribute to the strong enhancement of the S_{22} reflection signal (Fig. S13**b**, top row in Fig. S7) in addition to that of the magnon-phonon hybridization. However, the strong transmission S_{21} of mode X still mainly results from the magnon-phonon hybridization. This is because flat magnon mode has zero group velocity and magnon-phonon hybridized mode exhibits high group velocity and long life-time. One can also observe from Fig. S8 that the 50-50 hybridization point is on the tail of the driving field spectrum determined by the NSL width (=270 nm for the main data sets). This indicates that the most notable features of our measurements may originate from the region somewhat off the exact magnon-phonon crossing point. As we argued in the main text, the longest-propagating magnetic signal is likely to come from those only weakly hybridized with phonons.

Figs. S7 and S8 suggest that at $b_{\text{eff}} = 2 \times 10^5$, the transmission S_{21} can exhibit observable signatures of magnon-phonon hybridization while the anti-crossing gap itself is difficult to observe. For comparison, we simulated S_{21} at $d = 6\ \mu$m alongside $|\det \chi_k|$ at $\theta = \pi/4$ as a function of k for fixed $H = 100$ mT and as a function of H for fixed $k = 16$ rad/μm (Fig. S9). Note that the numerical constant c is a phenomenological fitting parameter so that we tried several values for each coupling strength and picked $c = 10^5, 2 \times 10^4, 6000, 1000, 1000$ respectively. As can be seen, $b_{\text{eff}} = 2 \times 10^5$ is optimal for reproducing the experimental S_{21} and the other values do not work. At $b_{\text{eff}} = 2 \times 10^4$, the anti-crossing gap becomes comparable with the linewidth. The signal is still visible only because one could indefinitely increase c to artificially enhance the mode X. $b_{\text{eff}} = 2 \times 10^6$ generates too large a hybridization for magnons far away from the crossing point. The signal decays faster in the interior of the mode X where the group velocity is significantly lower than 2.5 km/s. As far as anti-crossing is concerned, however, $b_{\text{eff}} = 2 \times 10^5$ does not make much of a difference from smaller values. To see anti-crossing gap of order GHz would require $b_{\text{eff}} = 2 \times 10^7$, which is totally unrealistic. Comparison with Fig. 4b also suggests that the CPW excitation is very much broadened and the observed S_{21} contains contributions from a broad range of k.

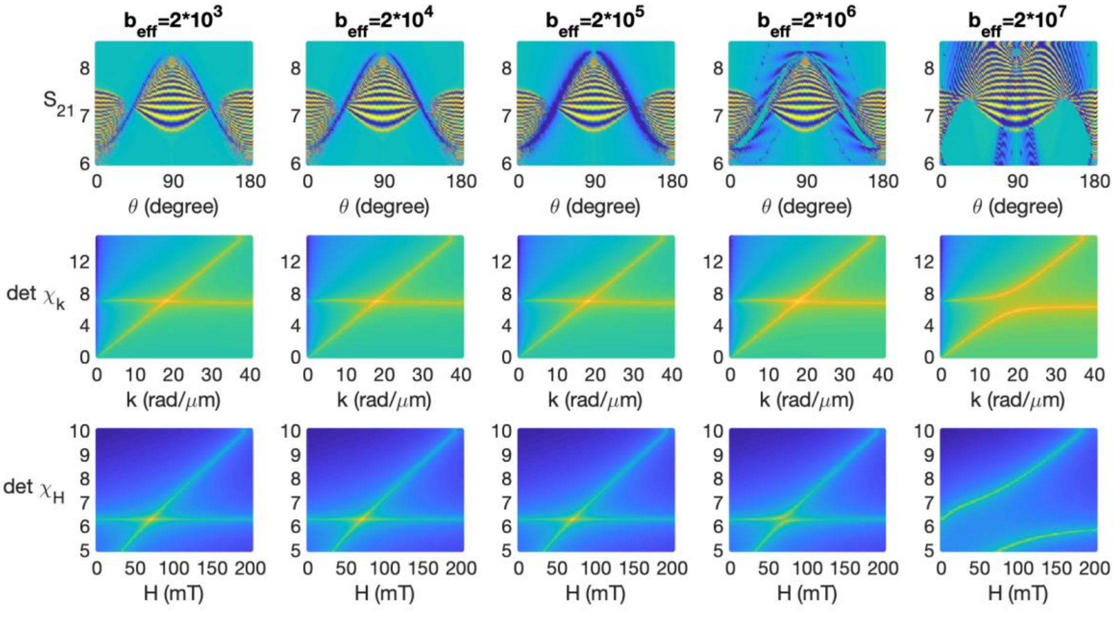

Fig. S9. The numerical results for different values of b_{eff}**.** Five representative values of b_{eff} from 2×10^3 to 2×10^7 were chosen, and S_{21} for $d = 6\ \mu$m (first row), $|\det \chi_k|$ as a function of k (second row) at $H = 100$ mT, and $|\det \chi_k|$ as a function of H (third row) at $k = 16$ rad/μm were calculated. Up to $b_{\text{eff}} = 2 \times 10^5$, the

observable difference is small and there is a significant arbitrariness in S_{21} in the choice of parameter c. Larger values of $b_{\rm eff}$ are definitely incompatible with the experimental results as the anti-crossing gap magnifies itself in S_{21}, however small we choose c to be.

Let us also mention the possibility of dissipative coupling between magnons and phonons. Although it would not be generated from the usual magneto-elastic coupling, it might arise, for instance, if there is another dynamical degree of freedom, i.e. other acoustic or spin wave modes, mediating the interaction between the LSMO magnons and BFO phonons. To see how dissipative coupling manifests itself, we simulated the case of extreme dissipative coupling where the susceptibility tensor in Eq. S3 is replaced by

$$\chi_{k\,\rm dissip}^{-1} = \begin{pmatrix} i\alpha M_s\omega/\gamma - F_{zz} & iM_s\omega/\gamma & 0 \\ -iM_s\omega/\gamma & i\alpha M_s\omega/\gamma - F_{\parallel} & -2ib_{\rm eff}\sin 2(\beta-\theta_M) \\ 0 & -2ib_{\rm eff}\sin 2(\beta-\theta_M) & \rho(\omega^2/k^2 - c_s^2 + i\omega/\tau k^2) \end{pmatrix}.$$

The results of S_{21} and $|\det\chi_k|$ are plotted in Fig. S10 for $b_{\rm eff} = 2\times10^5$ and $b_{\rm eff} = 2\times10^6$. As can be seen, for the realistic coupling, whether the coupling is conservative or dissipative would not make much of a difference. While dissipative coupling might give a minor improvement in the propagation distance, our experimental resolution is insufficient to discuss its relevance. Given that there is no concrete mechanism proposed for dissipative magnon-phonon coupling, we stick with the usual magneto-elastic coupling in this study.

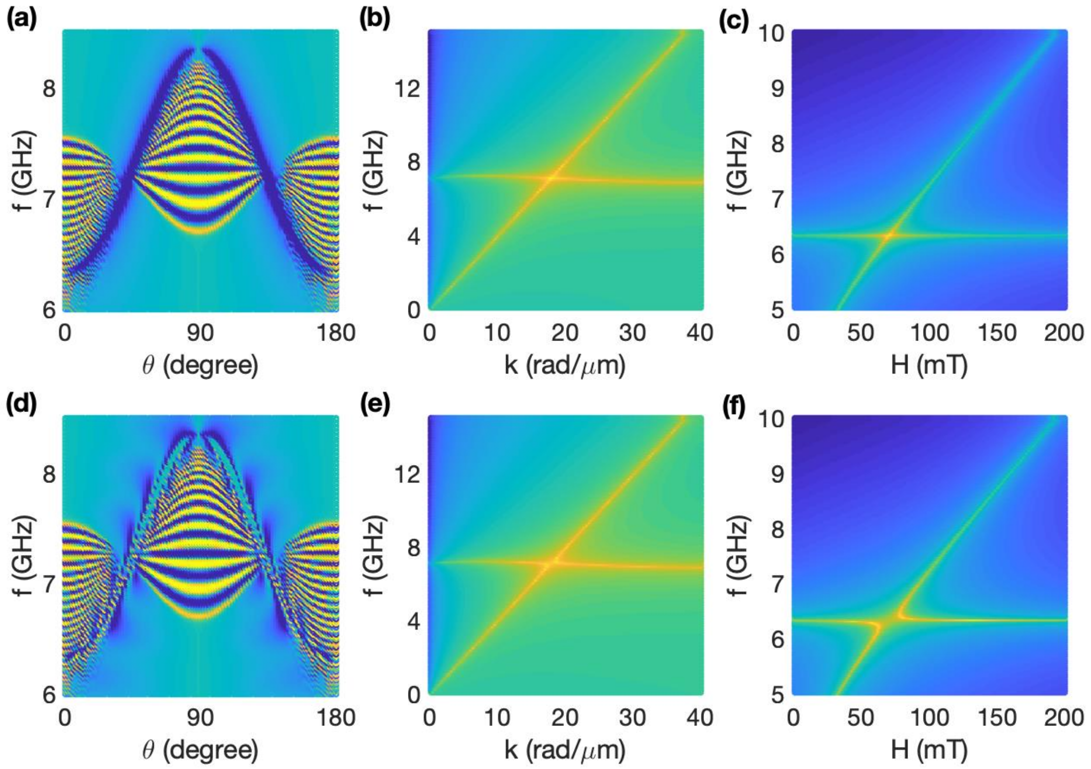

Fig. S10. The numerical results for dissipative couplings. (a-c) Simulations with $b_{\rm eff} = 2\times10^5$ for (a) S_{21} at $d = 6\ \mu$m, and $|\det\chi_k|$ (b) as a function of k at fixed $H = 100$ mT and (c) as a function of H at fixed $k = 16$ rad/μm. For this value of coupling, the difference from the ordinary coupling (Fig. S9) is hardly visible. **(d-f)** Simulations with $b_{\rm eff} = 2\times10^6$ for (d) S_{21} at $d = 6\ \mu$m where $c = 1000$, and $|\det\chi_k|$ (e) as a function of k at fixed $H = 100$ mT and (f) as a function of H at fixed $k = 16$ rad/μm. The level attraction can be observed for $|\det\chi_k|$ and S_{21} exhibits a different interference pattern from the non-dissipative case.

In relation to dissipative coupling, let us also remark that our experiment might also be analyzed in the light of input-output theory adopted in Ref. [51]. In this way, the coupling between the microwave

photon of NSL or CPW with magnons could be considered dissipative via intermediate phonon dynamics, for example. This approach, however, requires adding a dynamical degree of freedom to Eq. (S1), and completely alters the perspectives taken here. Since the magnon-phonon coupling theory with the microwave photon treated as an external driving already explains the experimental data satisfactorily, we leave the application of input-output theory for a future study.

And finally, let us briefly review how the propagation distance is related to the group velocity and the linewidth: We shall see that the intuitively obvious formula $\lambda = 2v_\mathrm{g}/\Delta\omega$ cannot easily be derived for dipolar spin waves. As we already noted, the coherent oscillation of the wave for a given frequency ω at a distance d from the source is given by

$$\begin{pmatrix} n_z(d) \\ n_\parallel(d) \\ \epsilon(d) \end{pmatrix} \propto \int dk\, \chi_k \begin{pmatrix} -\mu_0 M_\mathrm{s} h_k \\ 0 \\ f_k/k \end{pmatrix} e^{ikd}$$

in our setup. We replaced the sum over k by an integration. Without loss of generality, we take $d > 0$. Usually, χ_k as a function of k has a finite number of simple poles in the upper half plane corresponding to the resonant eigenmodes at the given frequency. In our case, there should be two such poles corresponding to the two degrees of freedom (there could be more, but they are ignored for clarity of presentation). For sufficiently small α, τ^{-1}, these poles should be close to two of the four real roots of Eq. S6 as an equation for k. Let k_1, k_2 be those real roots and q_1, q_2 the corresponding complex roots. The approximation to the complex roots is given by

$$q_1 \approx k_1 + i\frac{\Delta\omega_{k_1}}{2\partial\omega_{k_1}/\partial k_1}, q_2 \approx k_2 + i\frac{\Delta\omega_{k_2}}{2\partial\omega_{k_2}/\partial k_2},$$

where $\Delta\omega_k$ is the full width at half maximum for the resonance at k. Note that $\partial\omega_k/\partial k$ has to be positive for the roots to be in the upper-half plane, which picks out the two roots out of the four real ones. Particularly for the backward volume mode, $k < 0$ even though we calculate the amplitude at $d > 0$. Now one closes the contour in the upper-half plane and the application of the residue theorem yields

$$\begin{pmatrix} n_z(d) \\ n_\parallel(d) \\ \epsilon(d) \end{pmatrix} \propto \sum_{l=1,2} G_{q_l} \begin{pmatrix} -\mu_0 M_\mathrm{s} h_{q_l} \\ 0 \\ f_{q_l}/q_l \end{pmatrix} e^{iq_l d},$$

where G_q is a function independent of d. The decay length λ is determined by the imaginary part of $q_{1,2}$, yielding $\lambda = 2v_\mathrm{g}/\Delta\omega$. This derivation relies on the assumptions of analyticity of χ_k, though. For the thin-film approximation of magneto-static spin waves, $F_{zz}, F_\parallel$ are non-analytic functions of k through the dependance on $|k|$. Consequently, even if there are only four real roots of (S6), it is not straightforward to tell how many poles of χ_k exist in the upper-half plane. Moreover, the presence of branch cuts precludes the application of the residue theorem. Therefore, strictly speaking, it is unclear whether one could attribute the greater decay length of the magnons hybridized with phonons to the higher group velocity and lower relaxation rate. The numerical calculations presented in the previous section do not rely on analyticity and indicate that the hybridized mode indeed has a longer decay length than bare magnons. It appears to the authors that it is reasonable to expect that $\lambda = 2v_\mathrm{g}/\Delta\omega$ still gives a good approximation to the magnon decay length. The purpose of this paragraph was just to point out that a mathematical proof of this formula, in its simplest form at least, is not applicable here so that we cannot exclude the possibility that the observed long decay length could arise partially or entirely from a failure of the physical picture presented in V.

VII. Angle dependent spin-wave spectra of different *k* orientation

To determine the origin of mode A and mode B, we reoriented of the NSL antennas with respect to the [110] crystal direction of LSMO at 0°, 30°, 45°, 60°, 90°, the optical photo of the Chip Z and the full spin-wave transmission spectra of the different orientation devices are shown in Fig. S11. The spin-wave propagation distances are all fixed at 6 μm for all the data presented below. The magnitudes of magnetic field are all fixed at 100 mT. The orientation of the wave vector for each device is defined by the angle β as denoted in the optical photo of the sample in Fig. S11. In both the reflection and transmission spectra, we highlight two characteristic modes by fitted blue and red circles. β is the angle between the [110] crystal direction of the LSMO film and the wave vector k of each device as denoted in the optical photo of the sample. With the changing of β, mode A (blue circles) experiences a corresponding angle shift in its angular dependence while mode B (red circles) stays unchanged with respect to the [110] crystal direction of the LSMO film. Thus, mode A is attributed to the conventional magnon mode of the ferromagnetic LSMO layer while mode B is attributed to the layer adjacent to the anisotropic BFO/LSMO interface.

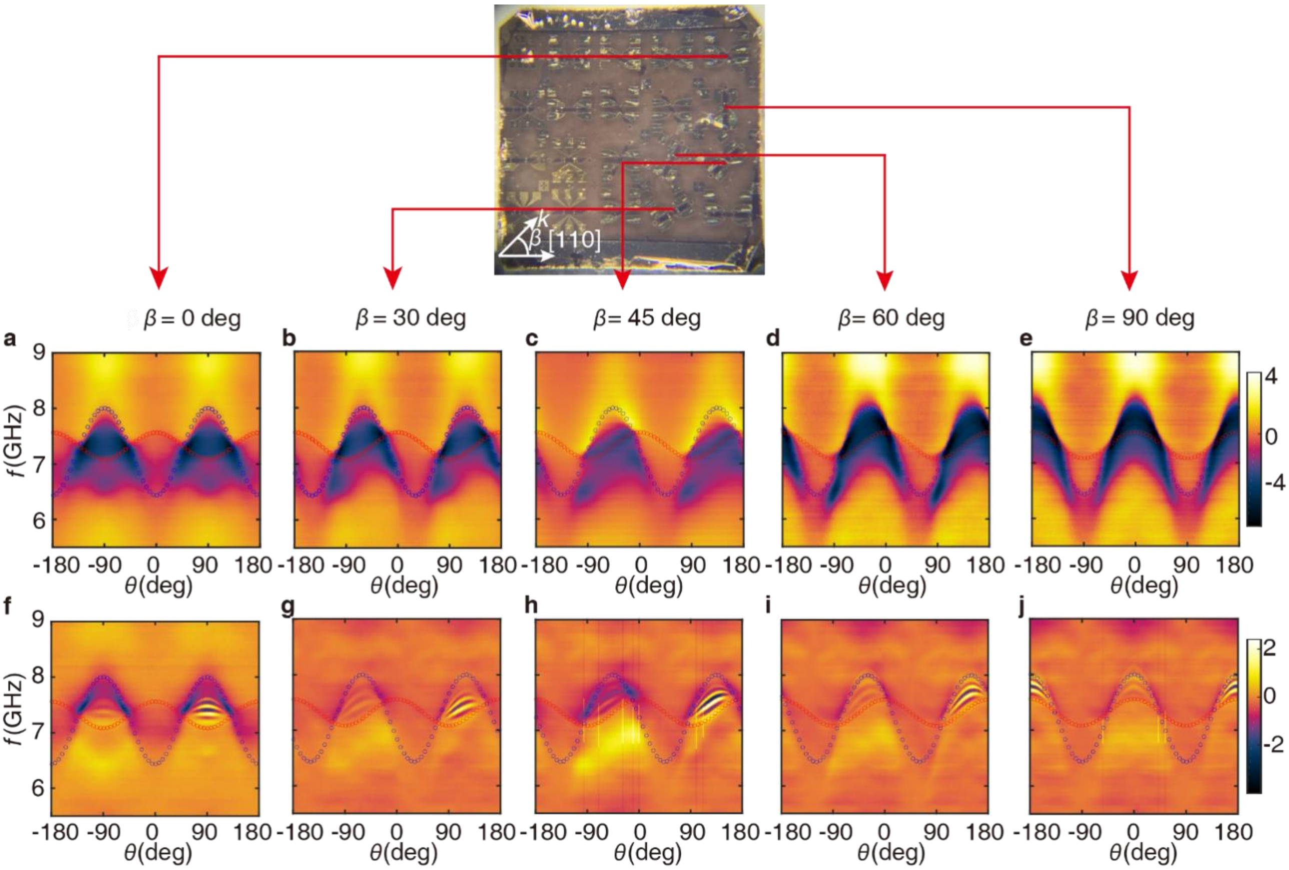

Fig. S11. Angle-resolved spin-wave spectra on sample Z taken on devices of different *k* orientations. a-e, Reflection spectra S_{22} taken on the devices of different orientations with respect to the [110] crystalline of the LSMO film. **f-j,** corresponding transmission spectra S_{21} of **a-e**. The external magnetic field is 100 mT for all the measurements displayed in this figure. The propagation distances of these devices are all fixed at 6 μm.

VIII. The scanning nitrogen-vacancy magnetometry characterization

The scanning nitrogen-vacancy magnetometry (NV) characterization of the LSMO/NGO sample and the BFO/LSMO/NGO sample are presented in Fig. S12. Panels in the top row (**a-c**) show measurements recorded on the LSMO surface of the LSMO/NGO sample, while measurements displayed in the bottom row panels (**d-f**), are recorded on the BFO surface of the BFO/LSM/NGO sample. In both cases magnetic stray-field imaging allows to clearly discern the stripe domain magnetic state, its regularity and orientation (Fig. S12**a** and Fig. S12**d**). More details are provided in the caption. Over the course of these experiments, a different behavior for these samples regarding the orientability of the remanent stripe domain state, when initializing the stripe domain texture from a high magnetic field state was observed.

Previous experiments on LSMO films showed that the application of an in-plane field above 100 mT followed by a subsequent decrease back to zero field results in a remanent magnetic state exhibiting stripe domains oriented along the selected field direction (Ref. 11 in the main text). In our experiments this reorientation could only be observed for the LSMO/NGO sample, while for the BFO/LSMO/NGO sample, the stripe domains remained oriented along the [110] crystal direction of LSMO. This observation provides an additional indication of the presence of an in-plane anisotropy in agreement with the in-plane anisotropy seen in the angular dependent spin-wave spectroscopy experiments, with its easy direction along the [110]-crystal direction.

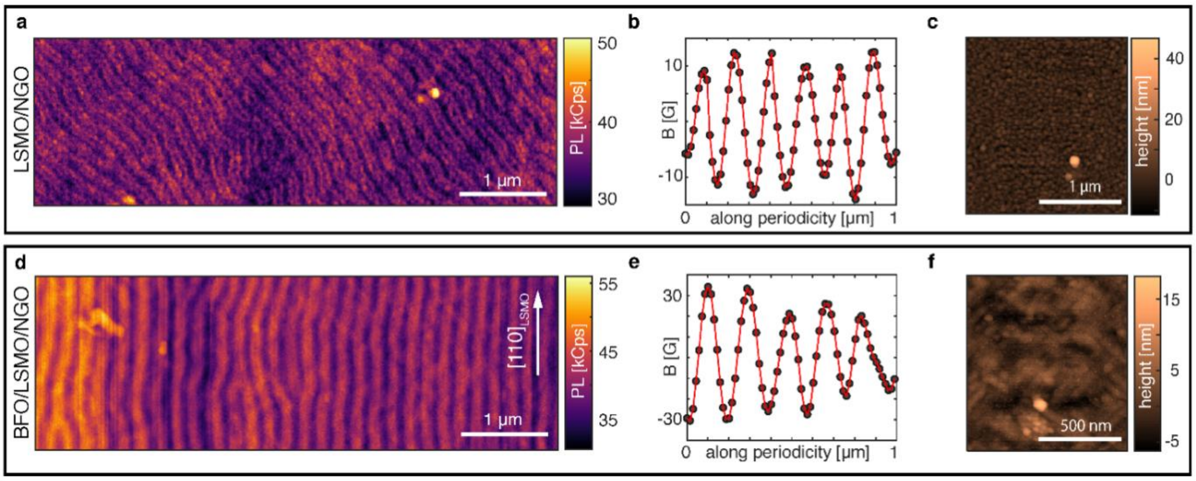

Fig. S12. Magnetic field mapping and topography of the LSMO film (top panels) and the BFO/LSMO film (bottom panels). **a**, **d**, Magnetic field maps recorded in 'quenching mode' sensitive to transversal fields with respect to the nitrogen vacancy axis. The stripe domain's stray field pattern, its orientation and degree of regularity is clearly discernible from the photoluminescence contrast. Its periodicity varies locally on the order of 10 nm with a mean value of 177 nm (LSMO) and 203 nm (BFO) respectively. The NV orientation has been previously determined to be φ=85°±5° (in-plane angle versus x-axis) and θ=50.7°±9° (out-of-plane angle away from the surface normal). **b**, **e** Stray field profiles across the stripe domain pattern as extracted from two-dimensional magnetic maps recorded with a sample-NV separation of nominally ~70 nm. From reference measurements its orientation is determined to be φ=90°±10° and θ=61°±5°. The periodicity matches the one observed in quantitative mode and a quantitative analysis yields for LSMO: 165 nm±20nm and for BFO 190 nm±20 nm. Detected fields are on the order of 10 G. The line profiles have been FFT filtered in the cutoff spatial frequency domain to remove measurement artifacts. **c**, **f**, topographic images showing the granular structure of the LSMO surface and the crystalline grains on the BFO surface. The surface roughness as estimated by the standard deviation of the height profile and its uncertainty is 3.07 nm±0.57 nm for the LSMO surface and 1.8 nm±0.76 nm for the BFO surface.

IX. Spin waves excited by meander coplanar waveguides

On the meander CPW (Sample Z15) with discrete wavevector distribution, we did the same angle resolved and the field resolved spin-wave measurement at the angle of $\theta = 54°$. The optical photo of the device is shown in Fig. S13**a**, the center-to-center distance of the two identical meander CPWs is 12 μm. The spin-wave reflection and transmission spectra of the angle dependence measurement are displayed in Fig. S13**b** and Fig. S13**c**. At the field angle of 54° where mode X is strongest in the NSL excited spectra, we did field scan measurement and the transmission spectra as shown in Fig. S13**d**. In the spectra, besides the magnetic field dependent magnon mode, whose frequency increases with the magnetic field, there are three discrete horizontal dark modes, whose frequencies are independent of magnetic field, labeled with k_1, k_2, k_3, are attributed to the phonon modes excited by the discrete wave vectors of the meander CPW as shown in Fig. S2**i**. The field independent mode is not obvious in **c** because we subtract the signal at 280 mT as background to reduce the noises.

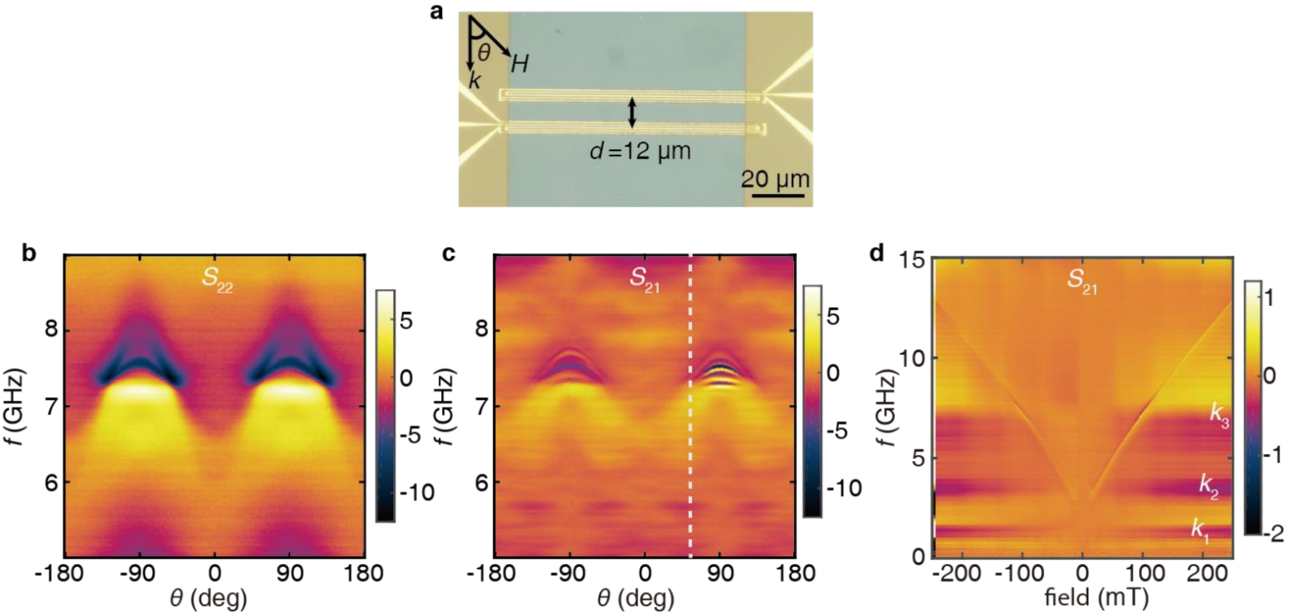

Fig. S13. Measurement results of using meander coplanar waveguides. a, The optical microscope image of the meander CPWs whose center-to-center distance is 12 μm. Scale bar, 20 μm. **b**, **c**, Angle resolved spin-wave reflection and transmission spectra excited by meander CPWs with propagation distance of 12 μm. **d**, Spin-wave spectra S_{21} obtained on meander CPW devices at $\theta = 54°$.

X. Phonon excitation by interdigital transducer on BFO film

Figure S14 shows the interdigital transducer (IDT) and its excited transmission spectra S_{21} on bare BFO film of 20 nm in thickness. We fabricated two pairs of identical IDT devices with the propagation distances of 50 μm and 1mm on the BFO film as shown in **a**, for each IDT, the width of the gold stripe is 600 nm with 2 μm between neighboring stripes, the number of period is 20. From the transmission spectra S_{21} displayed in **b**, we can observe clear dips at both distances, the transmission spectra decays not much over the distance of 1 mm. From a primitive exponential fitting, we can obtain a decay length of ~1.6 mm for the phonon signal in BFO film, which can confirm the long lifer time of the phonon modes in BFO film. From the linear fitting of the dispersion relation of the phonon mode in BFO film as shown in **c**, the group velocity of the phonon mode is estimated to be 3.2 ± 0.1 km s^{-1}. It is worth

noting that the thermal phonons excited in the experiments might play a role in the formation of the observed magnon-phonon hybridization mode with wide frequency range and relative higher frequency.

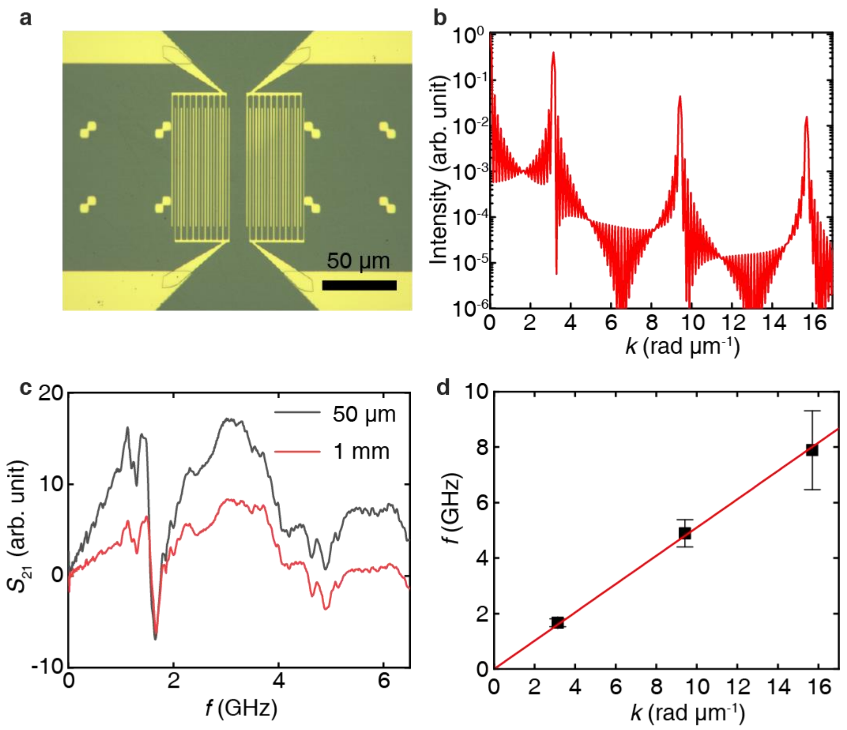

Fig. S14. IDT excited phonon propagation over long distances on pure BFO film. a, the optical picture of the interdigital transducer devices of the center-to-center distance $d = 50$ μm, the scale bar is 50 μm. **b**, k distribution of the IDT device obtained from Fourier transform according to its dimensions. **c**, The transmission signal of phonon over the propagation distances of 50 μm and 1 mm, the line-plots are obtained at the magnetic field of 0 mT. **d**, the linear fitting of the dispersion of the phonon mode in BFO film.

XI. Time resolved BLS measurement results at different propagation distances

To further confirm the long decay length of mode X, we did the time resolved BLS measurement at longer distances on Sample Z. The longest detection distance is 55 μm away from the NSL excitation antenna. The measurement was conducted at the same configuration as Fig. 3 in the main text. A microwave pulse with the frequency of 7.5 GHz is injected into the nano-stripline. Rising and falling edges can be observed by time-resolved BLS. From the lineplots of BLS signal obtained at different distances to the NSL antenna, different time delays at the rising and falling edges can be observed, which demonstrate the obtained signal is not induced by crosstalk, which propagates with the speed of light.

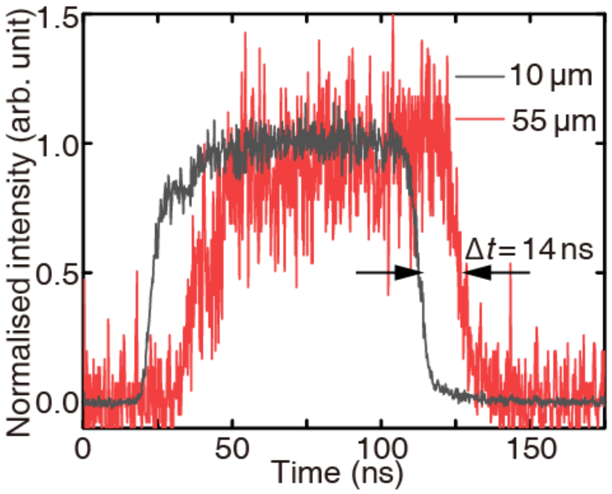

Fig. S15. Time-resolved BLS measurements at different distances. The measured positions are adjacent to the excitation NSL antenna with a distance of 10 μm and 55 μm away from the NSL antenna, respectively. The intensity of the BLS signal displayed in the figure are normalised to their maximum value for comparison. The measurements are conducted at 100 mT with the external field applied at 50° with respect to the [110] crystal direction of LSMO.

XII. Angle dependence of spin-wave group velocity

To have a clear look at the angle dependence of the spin-wave group velocity of mode Y, we extracted the group velocities of mode Y from Fig. 1**d** in the main text and plot the data as below.

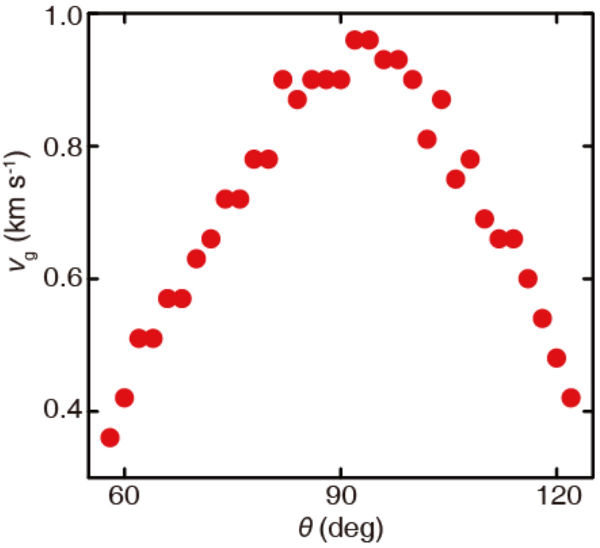

Fig. S16. Angle dependence of spin-wave group velocities. The red dots are spin-wave velocities of mode Y extracted from the transmission spectra S_{21} on Sample Z02 (d = 6 μm) shown in Fig. 1**d** in the main text. The wavevector k is parallel to the [110] crystalline orientation of LSMO.

XIII. Spin-wave transmission spectra of different propagation distances

Figure S17 presents the transmission spectra excited by the NSL antennas with different propagation distances (Samples Z01-02 and Z07-14). For example, the transmission signals of mode Y can be easily observed when the propagation distance is smaller than 24 µm. With the increasing of the propagation distance, the number of the signal oscillation is increased due to the phase accumulation during the process of propagation. While the strengths of mode Y become weaker contributed from the conventional decay phenomena, and become invisible when the propagation distance is larger than 24 µm. As for the mode X discussed in the main text, we always clearly observed them on different propagation distances devices. It is worthy to note that the mode X is still visible in 300 µm device, indicating larger diffusion length compared to mode Y.

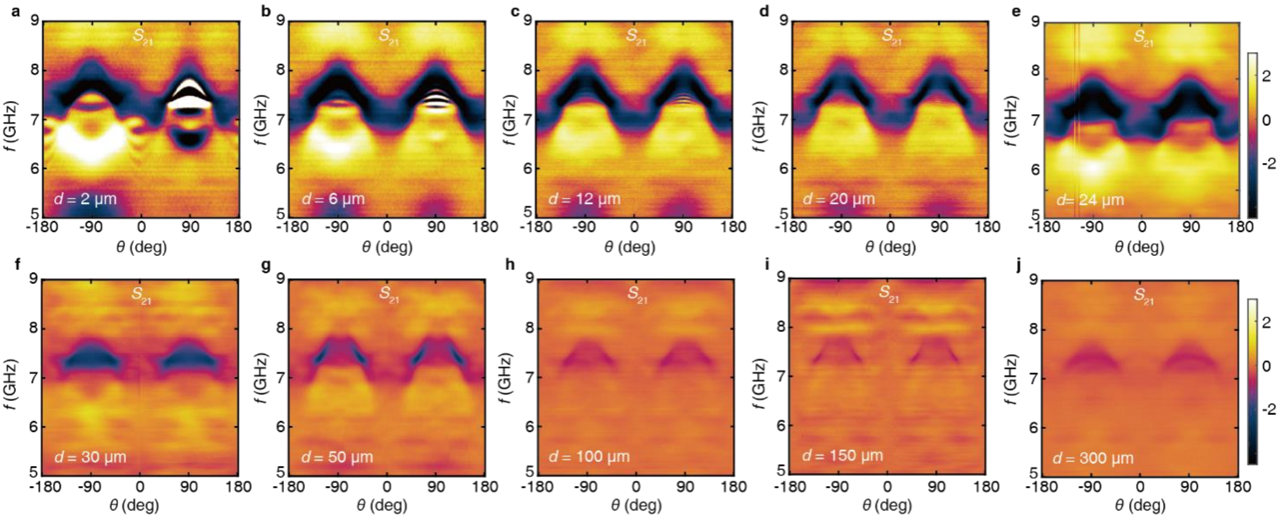

Fig. S17. Angle-resolved spin-wave transmission spectra S_{21} on Sample Z. The propagation distance d for each measurement is denoted on the bottom. From **a** to **j**, with the propagation distance increasing, mode Y near ±90° quickly fades away while the mode X near ±54°, ±126° sustains over a propagation distance of 300 µm.

Fig. S18 presents the field resolved spin-wave transmission spectra at the angle of 54° excited by the CPW antennas with different propagation distances (Sample N01-05). The SEM image of the device is shown in Fig. S18**a**. In the field scan spectra as shown in **b-f**, mode X could survive over the distance of 1060 µm. We note that the real CPW excitation spectrum is further broadened from Fig. S2**i** due to imperfections in fabrication, and spatially inhomogeneous profiles of the induced electromagnetic fields. Thus, the transmission signal above 100 mT and around 10 GHz may be associated with higher order excitation of the antennas.

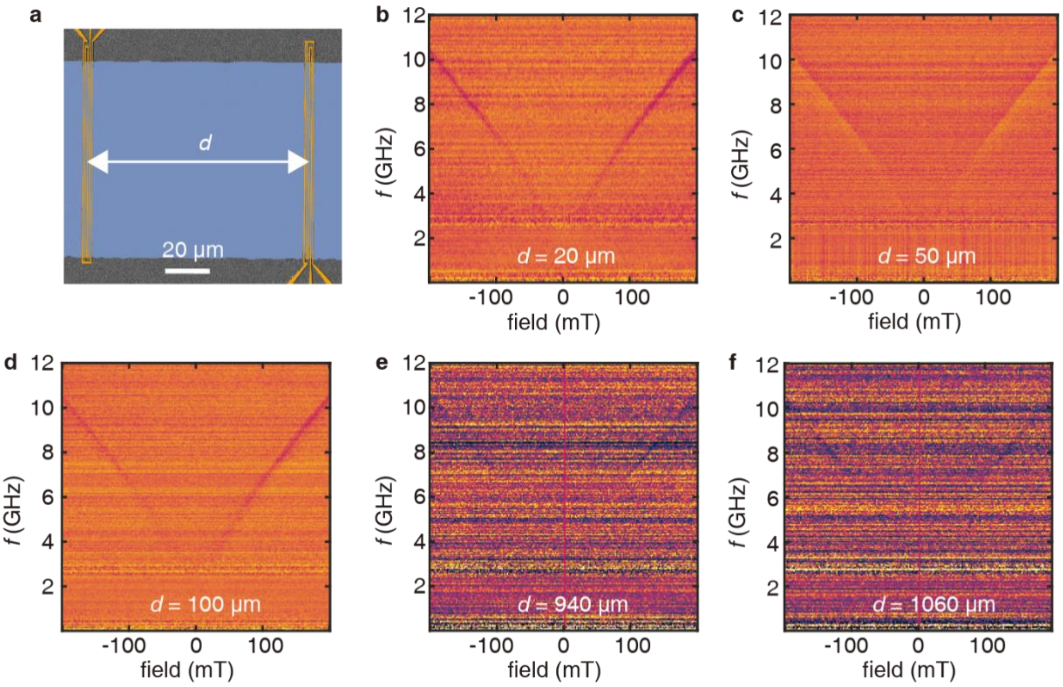

Fig. S18. Field resolved spin-wave spectra S_{21} obtained from meander CPW devices on Sample N series. a, the optical picture of the CPW devices of the center-to-center distance $d = 100$ μm, the scale bar is 20 μm. The propagation distances of each sample are denoted on the bottom of each spectra in **b-f**. The magnetic field was applied at $\beta = 54°$ with respect to the [110] direction of LSMO crystal. We can observe the spin-wave transmission signal over a propagation distance of 1060 μm.

XIV. Two-dimensional spatial map of mode X with micro-BLS

To characterize the spatial intensity distribution of mode X, we measured the 2D spatial map around the excitation antenna at the configuration of mode X. The 2D map of mode X along the stripline and perpendicular to stripline are shown in Fig. S19 and Fig. S20, respectively. The excitation frequency of the microwave injected into the antenna is fixed at 7.5 GHz while the external in-plane magnetic field of 100 mT is fixed at ~50° (49°) with respect to the [110] crystalline of LSMO for Fig. S19 (Fig. S20). From the angle resolved propagation spin wave measurement in Fig. 1**d**, we know at such configuration mode X is efficiently excited. With the micro-BLS we obtain the spatial map of mode X around the antenna. The yellowish bar at $y = 0$ μm denotes the stripline antenna used for magnon excitation, the hybridized mode propagates along the y direction.

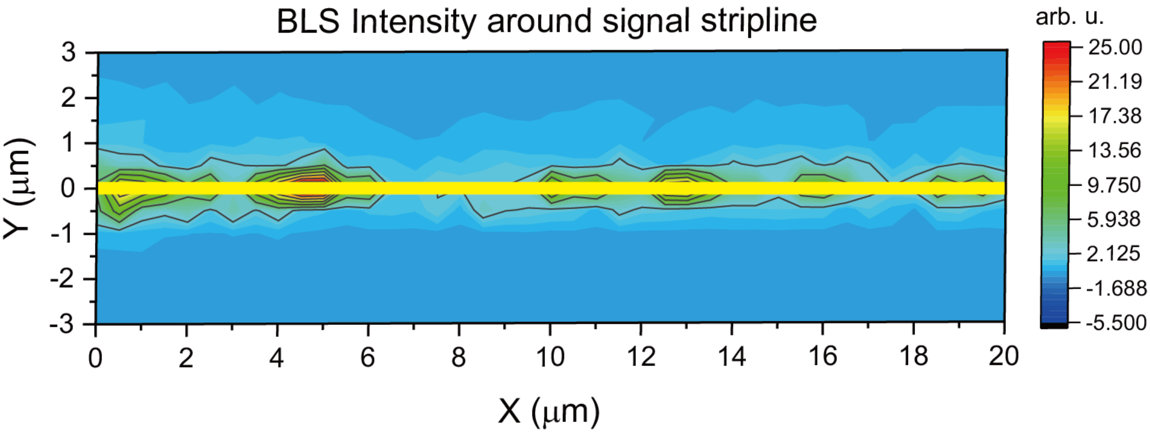

Fig. S19. Two-dimensional spatial map of mode X along the excitation antenna. The excitation microwave injected into the antenna, as denoted by the yellow stripe at $y = 0$, is fixed at 7.5 GHz, the external magnetic field of 100 mT is fixed at the angle of ~50°. At such configuration mode X is efficiently excited. The color in the map stands for the intensity of mode X.

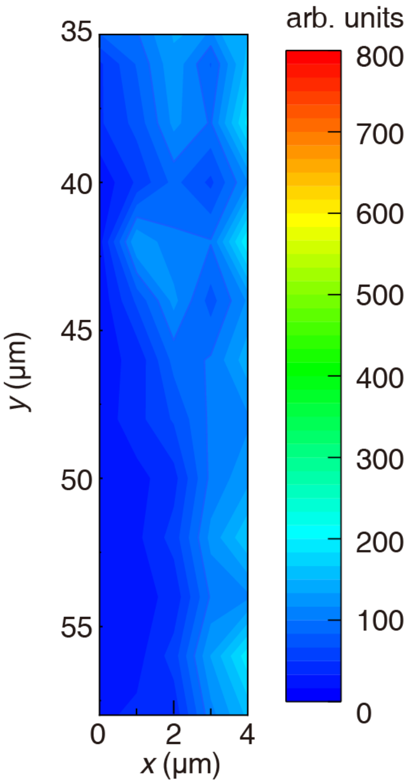

Fig. S20. Two-dimensional spatial map of mode X 35 μm away from the excitation area. The excitation microwave injected into the antenna at $y = 0$, is fixed at 7.5 GHz, and the external magnetic field of 100 mT is fixed at the angle of ~49°. At such configuration mode X is efficiently excited. The colors in the map stand for the intensity of mode X within the full measured window from $y = 35$ μm to 58 μm.

Reference

1. Ren, Y. H., Trigo, M., Merlin, R., Adyam, V. & Li, Q. Generation and detection of coherent longitudinal acoustic phonons in the $La_{0.67}Sr_{0.33}MnO_3$ thin films by femtosecond light pulses. *Appl. Phys. Lett.* **90**, 251918 (2007)

2. Landeros, P., Arias, R. E. & Mills, D. L. Two magnon scattering in ultrathin ferromagnets: The case where the magnetization is out of plane. *Phys. Rev. B* **77**, 214405 (2008).
