# Supplementary material for: Long decay length of magnon-polarons in BiFeO3/La0.67Sr0.33MnO3 heterostructures
Source: Nat Commun. 2021 Dec 14;12:7258. doi: 10.1038/s41467-021-27405-2 (PMC8671416; doi:10.1038/s41467-021-27405-2)
Supplement: Supplementary file 4 — Author Checklist [file 41467_2021_27405_MOESM4_ESM.pdf]

nature portfolio

Author Checklist

NCOMMS-NCOMMS-21-23224B

Please check the items below carefully and add a response in each row of the table to indicate the changes that you have made. Please also check through any additional marked-up edits we may have provided within the manuscript file.

Author information

Our guidance:	Your response:
Please review your complete author list to verify that it is complete and accurate. We ask that you consult with your coauthors to ensure that all names, affiliations, and titles are represented correctly. Note that if any authors are added or removed after this point then all authors will be requested to provide approval documentation that could potentially delay the production of your paper.	Checked

Article structure

Our guidance:	Your response:
We can accommodate up to 10 display items (Figures or Tables) in the main article. Each Figure and Table must fit easily within an A4 page (210 x 297 mm). Please ensure that the number and size of your Figures and Tables fulfil these requirements to avoid any delay in the acceptance of your article.	Checked
**Please ensure your main manuscript file includes the following sections, in this order:**<br>*Title*<br>*Author list*<br>*Affiliations*<br>*Abstract*<br>*Introduction*<br>*Results*<br>*Discussion (optional)*<br>*Results and Discussion (optional)*<br>*Methods (including Data Availability, Code Availability and Statistics subsections where relevant)*<br>*References*<br>*Acknowledgements*<br>*Author Contributions Statement*<br>*Competing Interests Statement*<br>*Tables*<br>*Figure Legends/Captions (for main text figures)*	Checked

We do not edit Supplementary Information files; they will be uploaded with the published article as they are submitted with the final version of your manuscript. Any tracked changes should be removed from the file and the file should be provided as a PDF file. Supplementary Figures do not need to be provided separately.	Checked

Main text

Our guidance:	Your response:
Please remove acronyms and abbreviations from the abstract.	Checked
Please make sure that mathematical terms throughout your manuscript and Supplementary Information (including in figures, figure axes, and legends) conform strictly to the following guidelines. Equations must be supplied in editable format, and not as images. Scalar variables (e.g. x, V, χ) must be typeset in italic, whereas multi-letter variables and functions (e.g. log) must be formatted in roman. Vectors (such as the wavevector k or the magnetic field vector B) must be typeset in bold without italics.	Checked

Figures and Tables

Our guidance:	Your response:
Please see the guidelines linked below for detailed instructions about how your figures should be prepared. Following these instructions will reduce the chances of delays should we need to request replacement artwork from you at a later stage.<br><br>https://www.nature.com/documents/NRJs-guide-to-preparing-final-artwork.pdf	Checked
All colour scales must be defined and intensity levels must be provided in either the figure or its associated legend. Please provide labels to these legends, e.g. at colour bars, in the format "variable [unit]" or similar.	Checked
Any abbreviations, symbols or colours present in your figures must be defined in the associated legends.	Checked
In each Figure and Supplementary Figure where error bars are used, they must be defined.	Checked

Data and Code

Our guidance:	Your response:
Nature journals strongly support public availability of data and code. Please deposit the data and code used in your paper into a public data repository, or alternatively, present the data as Supplementary Information. If data can only be shared on request, please explain why in your Data Availability Statement, and also in the correspondence with your editor.<br><br>Please note that for some data types, deposition in a public repository is mandatory. Any restrictions on sharing of these data	Checked

types must be clearly indicated in the statement and discussed with the editor. More information on our data deposition policies and available repositories can be found here: https://www.nature.com/nature-research/editorial-policies/reporting-standards#availability-of-data	
All published manuscripts reporting original research in Nature Portfolio journals must include a data availability statement, as a separate section before the References and under the heading 'Data Availability'.<br><br>The data availability statement must make the conditions of access to the “minimum dataset” that are necessary to interpret, verify and extend the research in the article, transparent to readers.<br><br>This minimum dataset may be provided through deposition in public community/discipline-specific repositories, custom proprietary repositories or general repositories like Figshare, Zenodo and Dryad. Providing large datasets in supplementary information is strongly discouraged and the preferred approach is to make data available in repositories. Scientific Data, a Nature Portfolio journal, maintains a list of approved and recommended data repositories to support researchers seeking suitable repositories for their data (https://www.nature.com/sdata/policies/repositories).<br><br>The Data Availability Statement should also reference any source data published alongside the paper.<br><br>If DOIs are provided, we also strongly encourage including these in the Reference list (authors, title, publisher (repository name), identifier, year).<br><br>For clinical datasets or third party data, please ensure that the statement adheres to our policy (https://www.nature.com/nature-research/editorial-policies/reporting-standards#availability-of-data)	Checked
Please use the following template to provide all the information stated above:<br><br>The XX data generated in this study have been deposited in the YY database under accession code ZZ [add hyperlink here]. The XX data are available under restricted access for {insert reason}, access can be obtained by {explain how}. The raw XX data are protected and are not available due to data privacy laws. The processed XX data are available at YY. The XX data generated in this study are provided in the Supplementary Information/Source Data file. The XX data used in this study are available in the YY database under accession code ZZ [Add hyperlink here].	Checked
An updated lasing reporting summary must be completed and uploaded as a supplementary information file with the revised manuscript. All points on the reporting summary must be addressed; if needed, please revise your manuscript in response to these points. This checklist is published alongside your manuscript online. Please note that this form is a dynamic "smart pdf" and must therefore be downloaded and completed in Adobe Reader, instead of opening it in a web browser. https://www.nature.com/authors/policies/Lasers.pdf	Checked

Methods

Our guidance:	Your response:
Sufficient details of the experiments must be provided in the Methods section such that they could be reproduced without reference to published papers. Use of the term "as described previously" is not encouraged.	Checked

References

Our guidance:	Your response:
Supplementary References should appear at the end of the Supplementary Information file, and must be self-contained and numbered from 1. References mentioned in both the main text and the Supplementary Information should be part of both reference lists so that the Supplementary Information does not refer to the reference list in the main paper and vice versa.	Checked

End matter

Our guidance:	Your response:
Please supply an "Author Contributions" section after the "Acknowledgements" section that refers to all authors. For more information on the Author Contributions statement, please refer to our authorship policy(https://www.nature.com/nature-research/editorial-policies/authorship), and to the following Nature Editorial: https://www.nature.com/articles/4581078a.	Checked
Nature Portfolio defines Competing Interest (CI) as financial and non-financial interests (including but not limited to funding, employment, stocks, shares, patents, personal or professional relationships with individuals or institutions, and unpaid membership advocacy) that could be perceived to directly undermine the objectivity, integrity, and value of a publication, or could be seen as having an influence on the judgments and actions of authors with regard to objective data presentation, analysis, and interpretation.<br><br>Please thoroughly review our policy on Competing Interests and include a detailed statement both in your final manuscript file and in our manuscript tracking system. Please ensure the statements are identical in both. Be specific about how each point stated relates to the research and list applicable author initials, and/or patent numbers.<br><br>If there are no competing interests, a negative statement must be included.<br><br>https://www.nature.com/nature-research/editorial-policies/competing-interests	Checked
Please confirm that all relevant funding awarded to each author is described in the Acknowledgements section. List each grant number, followed by the initials of the author who received it.	Checked

Preparing your manuscript files

Our guidance:	Your response:
Unless otherwise stated please limit individual file sizes to approximately 30MB. We strongly encourage the use of repositories for large datasets or source data due to size considerations.	Checked
Please supply a brief (maximum 250 characters, including spaces) summary of the main findings of the paper to be used on our website and in our e-alerts. The summary should be written in the third person in language suitable for a broad audience. The summary may be edited by the editors prior to publication. Please provide this summary in your cover letter.<br><br>Format of brief summary:<br>One sentence background. Here, the authors…	Provided in the cover letter.

Please supply the main Supplementary Information file as a single PDF file.	
The use or adaptation of previously published images is strongly discouraged. If this is unavoidable, please request the necessary rights documentation to re-use such material from the relevant copyright holders and return this to us when you submit your revised manuscript. Please check whether your manuscript or Supplementary Information contain third-party images, such as figures from the literature, stock photos, clip art or commercial satellite and map data.<br><br>For more information on what constitutes ownership by a third party, please contact our Editorial Assistant at naturecommunications@nature.com	Checked

Forms to complete

Our guidance:	Your response:
**Editorial Policy Checklist**<br>Please update and upload a final version of the Editorial Policy Checklist with your revised manuscript files. A blank Editorial Policy Checklist can be found via the link below. Note that this form is a dynamic 'smart pdf' and must be downloaded and completed in Adobe Reader.<br><br>Please update your current checklist or download from:<br><br>https://www.nature.com/documents/nr-editorial-policy-checklist.zip	Checked

You will need to upload:

Editorial Policy Checklist	Uploaded
Completed Third Party Rights Table (if relevant)	N/A
A point-by-point response to the reviewers' comments	Uploaded
A completed copy of this checklist	Uploaded
The main article file in Microsoft Word format - please supply a version with tracked changes and a version with tracked changes accepted	Uploaded
Separate Figure files	Uploaded
Inventory of Supporting Information	N/A
A Supplementary Information file	Uploaded
Lasing Reporting Summary	Uploaded
